# Experimental aerosol survival of SARS-CoV-2 in artificial saliva and tissue culture media at medium and high humidity

**DOI:** 10.1080/22221751.2020.1777906

**Published:** 2020-06-22

**Authors:** Sophie J. Smither, Lin S. Eastaugh, James S. Findlay, Mark S. Lever

**Affiliations:** Chemical Biological and Radiological Division, Defence Science and Technology Laboratory (Dstl), Wiltshire, UK

**Keywords:** SARS-CoV-2, coronavirus, survival, aerosol, saliva, humidity

## Abstract

SARS-CoV-2, the causative agent of the COVID-19 pandemic, may be transmitted via airborne droplets or contact with surfaces onto which droplets have deposited. In this study, the ability of SARS-CoV-2 to survive in the dark, at two different relative humidity values and within artificial saliva, a clinically relevant matrix, was investigated. SARS-CoV-2 was found to be stable, in the dark, in a dynamic small particle aerosol under the four experimental conditions we tested and viable virus could still be detected after 90 minutes. The decay rate and half-life was determined and decay rates ranged from 0.4 to 2.27 % per minute and the half lives ranged from 30 to 177 minutes for the different conditions. This information can be used for advice and modelling and potential mitigation strategies.

In late 2019 a novel coronavirus emerged in China that, within months, spread globally and has resulted in a world-wide pandemic. By early June 2020 over 7 million confirmed cases and over 400,000 deaths have occurred [1]. The pandemic has resulted in unprecedented restrictions on movement with much of the world in some form of lock-down to prevent spread. The causative agent of this pandemic is Severe Acute Respiratory Syndrome Coronavirus 2 (SARS-CoV-2). SARS-CoV-2 spreads through respiratory droplets. A recent study has shown the Washington variant of SARS-CoV-2 remains viable in a small particle aerosol for long periods [2]. Here we extend that research to look at a UK variant of SARS-CoV-2 in aerosols, at different relative humidity values, and in artificial saliva.

SARS-CoV-2 England-2 isolated from a 23-year-old male collected in January 2020 (GSAID Accession ID EPI_ISL_407073) was kindly provided by Public Health England. Passage 2 stocks were grown in Vero C1008 cells infected at MOI = 0·01, harvested after 3 days and clarified by centrifugation (350 g, 5 min in a Thermo-Scientific Sorvall Legend X1 centrifuge). Enumeration was via 50% tissue culture infectious dose (TCID_50_) assay: virus was ten-fold serially diluted in 96 well-plates of Vero C1008 cells and after 3–6 days at 37°C, wells at each dilution were scored for the presence of cytopathic effects by microscopic observation. Titre was determined by Reed & Muench calculation [3]. Tissue culture media (TCM) used was Dulbecco’s modified Eagle’s medium supplemented with 1% penicillin, streptomycin and l-glutamine.

Aerosol studies were conducted in a bespoke 40L Goldberg drum controlled by an AeroMP system (BiAera). Virus was aerosolised using a 3-jet Collison nebuliser and the drum was filled for 5 min at 30 L/min, sealed and mixed for 2 min to obtain a homogenous aerosol prior to sampling. Sampling was performed with midget impingers at 4L/min for 1 min into 3 mL TCM. Impinger counts were adjusted for dilution during sampling. Relative humidity (RH) was altered to achieve medium and high conditions and the aerosol was maintained at 19–22°C in the dark. For TCM studies, 8 mL virus was aerosolised. Artificial saliva was prepared according to the methods of Woo et al [4]. Virus was concentrated in an Amicon^®^ Ultra-15 centrifugal filter unit: 10 mL virus was centrifuged at 4000 g for 30 min and 70 µL concentrated virus was added to 5 mL artificial saliva prior to aerosolisation. Sprays for the four conditions were performed on three separate occasions and time-points were assayed in triplicate. Data was analysed in GraphPad Prism v 8.0.1. The exponential growth equation and least squares fit were applied to data sets.

SARS-CoV-2 England-2 variant was aerosolised in tissue culture media or in artificial saliva and maintained in a dynamic aerosol at medium RH (40–60%) or high RH (68–88%) (Supplementary Material: Table 1). The titre of virus in the aerosol was measured over time (Figure 1). All conditions started with similar titres (approximately 1 × 10^6^ TCID_50_/mL). TCM and artificial saliva both produced particles of 1–3 µm (measured using the Aerosol particle sizer APS 3321 [TSI inc.]) but overall, less artificial saliva particles were observed compared to particles of TCM (results not shown) accounting for the lower titres recovered (Figure 1). Viable virus could still be detected at 90 min under all experimental conditions. From replicate runs, best fit lines and decay rates were derived (Figure 1 and Supplementary Material: Table 1). In TCM, SARS-CoV-2 England-2 is more stable at medium RH (decay rate of 0·91% min^−1^) compared to higher RH (decay rate 1·59% min^−1^) whilst in artificial saliva the converse is true with a decay rate of 2·27% min^−1^ at medium RH and 0·40% min^−1^ at higher RH (Figure 1 and Supplementary Material: Table 1). Although the infectious dose in humans is not known, these experimentally derived decay rates suggest that if the virus is produced within small particle aerosols it may remain viable for at least 90 min. The results obtained with TCM at medium RH are similar to that already reported for the Washington variant of SARS-CoV-2 (half-life of 1·1–1·2 h [2] compared to our value of 1.25 h). We have extended the finding for SARS-CoV-2 to higher relative humidity and survival within artificial saliva. In TCM, SARS-CoV-2 England-2 survived less well at higher RH; similar observations have been reported for another coronavirus, MERS-CoV [5]. SARS-CoV-2 England-2 is more aerostable than the pathogenic filoviruses whose half-life was calculated as 15 min in the same system described here [6] and also twice as stable as Influenza virus at medium RH which gave a half-life of 32 min [7].

**Figure 1.**
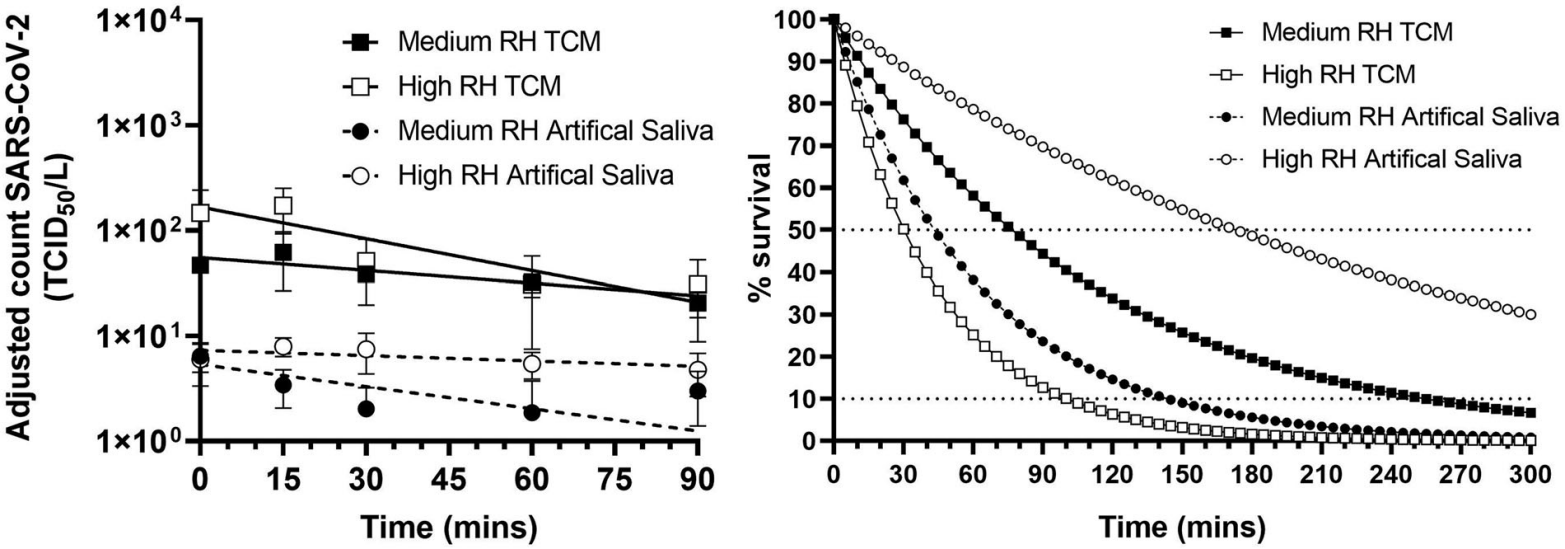
Survival of SARS-CoV-2 in a dynamic aerosol over time under different conditions. Left: SARS-CoV-2 was aerosolised in tissue culture media, TCM (squares) or artificial saliva (circles) and held in a Goldberg Drum for 90 mins at medium relative humidity, RH (filled shapes) or high RH (open shapes). Impinger samples were taken over time and enumerated by TCID_50_ assay. Each run was performed in triplicate and each time-point was assayed in triplicate. Counts were adjusted for dilution effect of sampling. Graph shows mean values (+/- SEM) for each condition with a line modelling exponential decay (solid lines for TCM, dashed lines for artificial saliva). Right: The theoretical decay of SARS-CoV-2 in TCM (squares, solid lines) or artificial saliva (circles, dashed lines) at medium RH (filled shapes) or high RH (open shapes) with decay as a percentage of starting amount shown. Horizontal dotted lines at 50% represent half-life and at 10% give an indication of time for starting amount to drop by 90% (1 x Log_10_).

Whilst coughing and sneezing generally produce large particles of saliva, smaller particles will also be produced, and small particles are also produced during routine activities such as talking [8] and breathing [9]. A recent study, for example estimated that 1 min of loud speaking could generate particles that could remain airborne for more than 8 min [10]. Another study has shown the 5 μm drops from the height of speaking or coughing take 9 min to reach the ground [11]. Smaller aerosol particles may be of concern because they may stay buoyant for longer, travel further and be able to penetrate further into the respiratory tract when inhaled. Medical procedures are also know to produce aerosols [12,13] so it is possible that aerosol particles of SARS-CoV-2 may be liberated during clinical interventions performed on patients infected with SARS-CoV-2. The data on the stability of SARS-CoV-2 in aerosols can be used to contribute to modelling (including transmission modelling) and advice on risk assessments and control measures. In summary, this study adds to our understanding of viral stability in small particle aerosols. Aerosols arising from human activity are heterogeneous in particle size and hence the data presented does not, on its own, directly inform likely risk of transmission.

## Supplementary Material

Supplemental Material

## References

[1] Coronavirus COVID-19 Global Cases. Available at: https://gisanddata.maps.arcgis.com/apps/opsdashboard/index.html#/bda7594740fd40299423467b48e9ecf6. Accessed 29 May 2020.

[2] van Doremalen N, Bushmaker T, Morris DH, et al. Aerosol and Surface stability of SARS-CoV-2 as compared with SARS-CoV-1. N Engl J Med. 2020;382(16):1564–1567. doi: 10.1056/NEJMc2004973 32182409 PMC7121658

[3] Reed LJ, Muench H. A simple method of estimating fifty percent endpoints. Am J Hygiene. 1938;27:493–497.

[4] Woo M-H, Hsu Y-M, Wu C-Y, et al. Method for contamination of filtering face piece respirators by deposition of MS2 viral aerosols. J Aerosol Sci. 2010;41:944–952. doi: 10.1016/j.jaerosci.2010.07.003 32226122 PMC7094656

[5] van Doremalen N, Bushmaker T, Munster VJ. Stability of Middle East respiratory syndrome coronavirus (MERS-CoV) under different environmental conditions. Euro Surveill. 2013;18:20590. doi: 10.2807/1560-7917.ES2013.18.38.20590 24084338

[6] Piercy TJ, Smither SJ, Steward JA, et al. The survival of filoviruses in liquids, on solid substrates and in a dynamic aerosol. J Appl Microbiol. 2010;109(5):1531–1539. 20553340 10.1111/j.1365-2672.2010.04778.x

[7] Schuit M, Gardner S, Wood S, et al. The influence of simulated sunlight on the inactivation of influenza virus in aerosols. J Infect Dis. 2020;221:372–378. doi: 10.1093/infdis/jiz582 31778532

[8] Asadi S, Wexler AS, Cappa CD, et al. Effect of voicing and articulation manner on aerosol particle emission during human speech. PLoS One. 2020;15(1):e0227699. doi: 10.1371/journal.pone.0227699 31986165 PMC6984704

[9] Papineni RS, Rosenthal FS. The size distribution of droplets in the exhaled breath of healthy human subjects. J Aerosol Med. 1997;10:105–116. doi: 10.1089/jam.1997.10.105 10168531

[10] Stadnytskyi V, Bax CE, Bax A, et al. The airborne lifetime of small speech droplets and their potential importance in SARS-CoV-2 transmission. Proc Natl Acad Sci U S A. 2020; May 13:202006874.

[11] Somsen GA, van Rijn C, Kooij S, et al. Small droplet aerosols in poorly ventilated spaces and SARS-CoV-2 transmission. Published Online May 27, 2020. DOI: 10.1016/S2213-2600(20)30245-9.

[12] Judson SD, Munster VJ. Nosocomial transmission of Emerging Viruses via aerosol-Generating Medical procedures. Viruses. 2019;11:E940. doi: 10.3390/v11100940 PMC6832307 31614743

[13] Davies A, Thomson G, Walker J, et al. A review of the risks and disease transmission associated with aerosol generating medical procedures. J Infect Prev. 2009;10:122–126. doi: 10.1177/1757177409106456

