# Supplementary material for: Experimental aerosol survival of SARS-CoV-2 in artificial saliva and tissue culture media at medium and high humidity
Source: Emerg Microbes Infect. 2020 Jun 22;9(1):1415–7. doi: 10.1080/22221751.2020.1777906 (PMC7473326; doi:10.1080/22221751.2020.1777906)
Supplement: Supplemental Material [file TEMI_A_1777906_SM1286.docx]

**Supplementary Material**

**Table 1. Experimental and decay parameters for aerosolised SARS-CoV-2 under different conditions**

|  | **Medium Relative Humidity (RH)** | | **High Relative Humidity (RH)** | |
| --- | --- | --- | --- | --- |
| **Spray Media^a^** | **TCM** | **AS** | **TCM** | **AS** |
| **Mean Collison count post-aerosolisation**  **(TCID_50_/mL)** | 1·11 x 10^6^ | 2·28 x 10^6^ | 1·63 x 10^6^ | 7·71 x 10^5^ |
| **RH during drum fill**  **(%)** | 54–57 | 53–57 | 84–88 | 80–82 |
| **RH Range during Impinger sampling**  **(%)** | 41–50 | 42–46 | 70–76 | 68–75 |
| **Decay Rate**  **(% min^-1^)** | 0·91 | 1·59 | 2·27 | 0·40 |
| **Half-Life ^b^**  **(mins)** | 75 | 42 | 30 | 177 |

^a^ TCM: Tissue culture media; AS: Artificial Saliva

^b^ Time taken for initial amount to decrease by 50%
